# Psychosocial Determinants of Non-adherence to Antihypertensive Therapy: A Cross-Sectional Study in Pakistani Tertiary Care Hospitals

**DOI:** 10.7759/cureus.79862

**Published:** 2025-02-28

**Authors:** Syed Shahzaib Ali, Muhammad Arslan Riaz, Muskan Fatima, Sabeen Arjumand, Maira Bhatti, Zarish Ghafoor, Fahad R Khan

**Affiliations:** 1 Department of Medicine, Punjab Rangers Teaching Hospital, Lahore, PAK; 2 Department of Pharmacology, Sharif Medical and Dental College, Lahore, PAK; 3 Department of Pharmacology, Sharif Medical and Dental Colege, Lahore, PAK; 4 Department of Cardiology, Lady Reading Hospital, Peshawar, PAK

**Keywords:** antihypertensive therapy, health literacy, hypertension, medication adherence, non-adherence, psychosocial determinants, public health, social support, stress

## Abstract

Background

Hypertension is a major public health concern and leading cause of cardiovascular morbidity and mortality worldwide. Non-adherence to antihypertensive therapy is a global challenge, with adherence rates ranging from 50% to 70% in high-income countries and significantly lower in low- and middle-income countries (LMICs). Poor adherence contributes to inadequate blood pressure control and increases the risk of stroke, myocardial infarction, and other cardiovascular complications. Even though effective antihypertensive drugs are available, adherence is still not very good, especially in LMICs because of problems with money, healthcare access, and psychosocial factors.

Pakistan, like many LMICs, faces a high burden of hypertension; however, adherence rates remain underreported due to inconsistent methodologies and a lack of large-scale studies. Psychosocial factors, including perceived stress, social support, and health literacy, play a crucial role in influencing medication adherence; however, limited research has explored these determinants within the Pakistani population.

Objective

This study aimed to investigate the psychosocial determinants of non-adherence to antihypertensive therapy among patients receiving treatment at tertiary care hospitals in Pakistan. By identifying the key psychosocial factors affecting adherence, this study sought to provide evidence-based recommendations for improving hypertension management strategies.

Methods

A cross-sectional study was conducted at three tertiary care hospitals in Pakistan: Punjab Rangers Teaching Hospital, Lahore; Sharif Medical and Dental College, Lahore; and Lady Reading Hospital, Peshawar. In total, 360 patients with hypertension were recruited for this study. Medication adherence was assessed using a Medication Adherence Questionnaire (MAQ). Psychosocial determinants were evaluated using validated scales, including the Depression, Anxiety, and Stress Scale-21 (DASS-21) for perceived stress; the Multidimensional Scale of Perceived Social Support (MSPSS) for social support; and the Living with Medicines Questionnaire (LMQ) for health literacy. Chi-square tests and multivariate logistic regression were used for statistical analysis to find independent predictors of non-adherence.

Results

Among the 360 participants, 145 (40.3%) were classified as nonadherent. Significant associations were observed between non-adherence and lower educational levels (87, 60.0%; p < 0.001), low social support (62, 42.8%; p < 0.001), high perceived stress (127, 87.6%; p < 0.001), and lower monthly income (79, 54.5%; p = 0.002). The most commonly reported reasons for non-adherence were forgetfulness (67, 46.2%), medication costs (52, 35.9%), and perceived lack of necessity (32, 22.1%). Hospital-wise adherence rates varied significantly, with the highest adherence at Punjab Rangers Teaching Hospital (119, 66.1%) and the lowest at Lady Reading Hospital (38, 52.3%) (p = 0.028).

Conclusion

Non-adherence to antihypertensive therapy remains a significant public health concern, particularly in LMICs, such as Pakistan, where financial constraints, low education levels, and psychosocial stressors impact adherence. Taking these factors into account through patient education, financial aid programs, and psychosocial support systems may increase the number of people who stick to their treatment plans and lead to better management of hypertension.

## Introduction

Hypertension is a leading global public health concern and a significant contributor to cardiovascular morbidity and mortality [1]. Despite the widespread availability of effective antihypertensive medications, adherence to prescribed therapy remains suboptimal, particularly in low- and middle-income countries (LMICs) such as Pakistan [2]. In Pakistan, medication adherence is influenced by unique sociocultural, economic, and healthcare system challenges that differentiate it from other regions [3]. The healthcare system is fragmented with limited access to specialists, high out-of-pocket medical expenses, and inconsistent primary care services, all of which contribute to poor adherence rates [4].

Non-adherence to antihypertensive therapy is a multifaceted issue shaped by patient beliefs, economic constraints, social dynamics, and healthcare accessibility [5]. While previous research has largely focused on pharmacological management and biomedical determinants, there remains a notable gap in exploring the psychosocial determinants that significantly impact medication adherence. Psychosocial factors, such as perceived stress, social support, and health literacy, play a critical role in determining adherence behaviors. Perceived stress, often heightened by financial difficulties and healthcare inaccessibility, has been associated with negative health behaviors, including inconsistent medication use [6]. Social support, including encouragement from family members and effective physician-patient interactions, has been shown to reinforce treatment importance and enhance medication compliance [7]. Moreover, inadequate health literacy, including misconceptions about hypertension and medication side effects, frequently leads to incorrect medication use or treatment discontinuation [8].

Previous studies have identified factors such as forgetfulness, lack of awareness regarding hypertension complications, and misconceptions about medication side effects as primary barriers to adherence [9]. However, limited research has explored psychosocial determinants within the Pakistani healthcare context. Existing studies often lack validated adherence measures and fail to examine the role of cultural and religious beliefs in shaping patient behaviors [10]. In Pakistan, religious and cultural influences play a significant role in health care decisions. Some individuals perceive hypertension as a transient condition that can be managed through faith-based healing or traditional medicine rather than lifelong pharmacological treatment [11]. This perception underscores the need for a more nuanced understanding of how cultural and religious perspectives intersect with adherence behaviors.

This study aimed to investigate the psychosocial determinants of non-adherence to antihypertensive therapy among patients receiving treatment at tertiary care hospitals in Pakistan. By addressing existing research gaps, including the lack of validated adherence measures and a limited focus on psychosocial determinants, this study provides an evidence-based framework to improve patient compliance. These findings have direct implications for clinical practice and public health policy, particularly in LMICs with similar healthcare and socioeconomic challenges. Understanding these factors will help in creating targeted interventions, such as patient education programs, social support initiatives, and stress management strategies, to improve adherence rates and, in the end, lower the burden of uncontrolled hypertension [12].

## Materials and methods

Study design and ethical considerations

This cross-sectional study was conducted at three tertiary care medical centers in Pakistan: Punjab Rangers Teaching Hospital, Lahore; Sharif Medical and Dental College, Lahore; and Lady Reading Hospital, Peshawar. These hospitals were selected based on their tier levels as tertiary referral centers, representing both public and private healthcare systems that cater to diverse socioeconomic groups. Punjab Rangers Teaching Hospital and Lady Reading Hospital serve as public referral centers providing subsidized care, while Sharif Medical and Dental College represents a private teaching hospital with different patient demographics. The study spanned from January 1, 2023, to December 31, 2023, ensuring the inclusion of patients from urban and semi-urban settings, reflecting diverse adherence behaviors across varying healthcare infrastructures. However, rural populations were not directly represented, which is a limitation of this study.

Ethical approval was obtained from the Institutional Review Boards (IRBs) of all participating hospitals: Lady Reading Hospital Medical Teaching Institute (approval No: 533/LRH/MTI/22), Punjab Rangers Teaching Hospital (approval No: 395/PRTH/22), and Sharif Medical and Dental College (approval No: 76/IRB/SMDC/22). Written informed consent was obtained from all participants to ensure their voluntary participation and confidentiality. This study adhered to the principles outlined in the Declaration of Helsinki.

Author contributions and logistics

This study was conducted through collaborative efforts among all authors. The principal investigator conceptualized the study design and secured ethical approval. The co-authors contributed to the data collection, patient recruitment, statistical analysis, and manuscript preparation. A designated research team at each hospital, led by a co-author, was responsible for overseeing daily study operations, ensuring protocol compliance, and maintaining data accuracy. Regular virtual and in-person meetings were held at all study sites to monitor progress, resolve challenges, and ensure consistency in data collection. Data entry and validation were conducted centrally, and the authors collectively reviewed the preliminary findings before the final analysis. A biostatistics expert performed the statistical analyses and collaborated with the authors to interpret the results and refine the conclusions. The final manuscript was reviewed and approved by all the authors to ensure the integrity and rigor of the study.

Participant selection

Patients were recruited from the outpatient and inpatient medical wards of participating hospitals. The inclusion criteria were as follows: (i) adults (≥18 years) with a confirmed diagnosis of hypertension for at least one year, (ii) an active prescription for antihypertensive medications, and (iii) willingness to participate. Patients with severe uncontrolled psychiatric conditions were excluded; however, individuals with managed anxiety or depression were included to ensure comprehensive evaluation of psychosocial determinants. The final sample size was 360 participants, proportionally distributed across the three hospitals based on patient volume.

Sample size determination

The sample size was determined using the WHO sample size calculator for cross-sectional studies, assuming a 30% prevalence of non-adherence to antihypertensive medications. This assumption was based on regional studies reporting similar adherence rates among patients in Pakistan [13,14]. A power calculation (80% power, 5% margin of error, 95% confidence level) yielded the required sample of 327 patients. The final sample size was set at 360 participants to account for a 10% non-response rate.

Medication adherence assessment

Medication adherence was assessed using the Medication Adherence Questionnaire (MAQ), a validated four-item scale widely used in South Asian populations [15]. The MAQ has been used to check how well people follow their high blood pressure treatment over time, and is very reliable (Cronbach's α = 0.79-0.82) in studies conducted in LMICs [15]. For linguistic and cultural appropriateness, the MAQ was translated into Urdu using forward-backward translation by bilingual experts. Pilot testing of a subset of 30 patients confirmed their comprehension and conceptual equivalence. Non-adherence was defined as a response of “yes” to at least two of the four items that assessed behaviors such as forgetting medication, discontinuing due to improvement, stopping medication when feeling worse, and skipping doses due to inconvenience [15]. Since self-reported adherence assessments are prone to recall and social desirability biases, patients were reassured that their responses would remain confidential and would not affect their medical care. Additionally, trained interviewers used neutral phrasing and follow-up prompts to minimize bias and ensure accurate responses.

Psychosocial determinants assessment

Psychosocial determinants were assessed using validated instruments. Perceived stress was measured using the Depression, Anxiety, and Stress Scale-21 (DASS-21), with total scores categorized as mild, moderate, or severe [16]. Social support was evaluated using the Multidimensional Scale of Perceived Social Support (MSPSS), where scores below 40 indicate low support, 41-60 indicate moderate support, and above 60 indicate strong perceived support [17]. Health literacy was assessed using the Living with Medicines Questionnaire (LMQ), with scores below 50 classified as inadequate medication literacy [18]. These instruments have been previously validated in Pakistan to ensure their cultural and contextual relevance.

Data collection and reliability measures

Data were collected through structured interviews and review of medical records. Interviews were administered in Urdu by trained medical officers and postgraduate trainees who received specialized training in standardized data collection to minimize inter-observer variability. Inter-rater reliability checks were conducted at each hospital by randomly reviewing 10% of participants’ responses. The data were entered into a centralized database with double-entry validation to ensure accuracy. Regular investigator meetings and audits maintained standardization across the study sites.

Medication regimes and classification

Patients were categorized into monotherapy and combination therapy groups based on their antihypertensive regimen. The study looked at both patients who were only taking one drug (like angiotensin-converting enzyme (ACE) inhibitors and beta-blockers) and those who were taking more than one drug. This is because many times, taking more than one drug is needed to effectively control blood pressure.

Statistical analysis

Data were analyzed using SPSS Statistics version 26.0 (IBM Corp., Armonk, NY, USA). Descriptive statistics, including means, standard deviations, and frequencies, were used to summarize the demographic and clinical characteristics. The chi-square test was used for categorical variables, and independent t-tests were used for continuous variables. Multivariate binary logistic regression was performed to identify independent predictors of non-adherence, adjusting for age, sex, education, income, and comorbidities. Model validation was conducted using the Hosmer-Lemeshow goodness-of-fit test, and Bonferroni corrections were applied to account for multiple comparisons. Results are presented as odds ratios (ORs) with 95% confidence intervals (CIs).

Handling of missing data and non-responders

Missing data were managed using multiple imputations for cases in which the adherence responses were incomplete. Patients who declined participation or withdrew consent were recorded as non-responders but were not included in the final analysis. Hospital-based psychosocial support referrals were recommended for participants exhibiting high psychosocial distress (severe stress or low social support).

## Results

A total of 360 patients diagnosed with essential hypertension were included in this study. The mean age of the participants was 56.2 ± 11.8 years, with 190 (52.8%) males and 170 (47.2%) females. The median duration of hypertension was eight years (IQR: 4-12 years). The majority of the participants, 221 (61.4%), resided in urban areas (including peri-urban regions), while 139 (38.6%) were from rural areas. The mean body mass index (BMI) was 27.3 ± 4.5 kg/m². Based on BMI classification, 98 (27.2%) participants were classified as normal weight (BMI < 25 kg/m²), 142 (39.4%) as overweight (BMI 25-29.9 kg/m²), and 120 (33.3%) as obese (BMI ≥ 30 kg/m²). Baseline demographic and clinical characteristics of the study population are presented in Table 1.

**Table 1:** Baseline characteristics of study participants Statistical tests conducted: T-test for continuous variables and the chi-square test for categorical variables. *A p-value < 0.05 is considered statistically significant. PKR: Pakistani rupee

Variable	Adherent, n (%)	Non-adherent, n (%)	p-value
Age (years, mean ± SD)	55.8 ± 11.5	56.7 ± 12.2	0.451
Male	115 (53.5)	75 (51.7)	0.732
Urban residence (including peri-urban)	138 (64.2)	83 (57.2)	0.198
BMI (kg/m², mean ± SD)	26.9 ± 4.3	27.8 ± 4.7	0.164
BMI category			
Normal weight (< 25 kg/m²)	67 (31.2)	31 (21.4)	0.035*
Overweight (25-29.9 kg/m²)	81 (37.7)	61 (42.1)	0.412
Obese (≥ 30 kg/m²)	67 (31.2)	58 (40.0)	0.091
Hypertension duration in years, median (IQR)	7 (3-11)	9 (5-13)	0.022*
Diabetes mellitus	85 (39.5)	68 (46.9)	0.211
Dyslipidemia	91 (42.3)	74 (51.0)	0.104
Smoking	42 (19.5)	57 (39.3)	<0.001*
Monthly income < 50,000 PKR	78 (36.3)	79 (54.5)	0.002*
Education level < high school (includes no formal education)	64 (29.8)	87 (60.0)	<0.001*
Social support score (mean ± SD)	28.7 ± 5.3	22.5 ± 6.1	<0.001*

A chi-square test was performed to evaluate the association between categorical variables and adherence status. Significant associations were observed for smoking (χ² = 15.12, p < 0.001), lower education levels (including individuals with no formal education) (χ² = 23.45, p < 0.001), and low social support (χ² = 19.87, p < 0.001). For the analysis, education level was dichotomized into "< high school (including no formal education)" vs. "≥ high school". Similarly, social support was categorized as "low" vs. "moderate/high" based on a cut-off score determined from the study population's distribution. The expected vs. observed frequencies for each categorical variable are provided in Table 2, offering further insight into the strength of these associations.

**Table 2:** Chi-square analysis of categorical variables and non-adherence *A p-value < 0.05 is considered statistically significant.

Variable	Adherent (observed/expected)	Non-adherent (observed/expected)	Chi-square (χ²)	p-value
Smoking	42/57.9	57/41.1	15.12	<0.001*
Education < high school (including no formal education)	64/87.6	87/63.4	23.45	<0.001*
Low social support	102/123.9	118/96.1	19.87	<0.001*

Among the 145 (40.3%) non-adherent patients, the most frequently cited reasons for non-adherence were forgetfulness (67, 46.2%), financial constraints (52, 35.9%), and perceived lack of necessity for medication (32, 22.1%). Other notable reasons included fear of side effects (28, 19.3%), lack of social support (26, 17.9%), complexity of the medication regimen (18, 12.4%), and cultural or religious beliefs (14, 9.7%). These findings are summarized in Table 3.

**Table 3:** Distribution of reasons for non-adherence Statistical test conducted: Frequency distribution

Reason for non-adherence	Frequency, n (%)
Forgetfulness	67 (46.2)
Financial constraints	52 (35.9)
Perceived lack of necessity	32 (22.1)
Fear of side effects	28 (19.3)
Lack of social support	26 (17.9)
Complexity of medication regimen	18 (12.4)
Cultural or religious beliefs	14 (9.7)

To further explore potential underlying patterns, subgroup analyses were conducted to assess whether certain demographic characteristics were associated with specific reasons for non-adherence. A chi-square test revealed a significant association between forgetfulness and older age (≥60 years) (χ² = 10.24, p = 0.001), suggesting that cognitive decline or age-related memory issues may contribute to medication non-adherence. Similarly, financial constraints were more frequently reported by patients with monthly incomes below 50,000 Pakistani rupee (PKR) (χ² = 8.93, p = 0.002) and those with lower educational attainment (<high school, including no formal education) (χ² = 7.65, p = 0.004). This finding indicates that socioeconomic barriers play a critical role in medication adherence, emphasizing the need for financial assistance programs or cost-effective treatment options for this patient population. Additionally, lack of social support was significantly correlated with patients who lived alone (χ² = 9.41, p = 0.002), highlighting the importance of community and family involvement in promoting adherence to antihypertensive therapy. This is illustrated in Table 4.

**Table 4:** Subgroup analysis of non-adherence by age and socioeconomic factors Statistical test conducted: Chi-square test. *A p-value < 0.05 is considered statistically significant. PKR: Pakistani rupee

Factor	Adherent, n (%)	Non-adherent, n (%)	Chi-square (χ²)	p-value
Age ≥60 years	58 (26.9)	65 (44.8)	10.24	0.001*
Monthly income < 50,000 PKR	78 (36.3)	79 (54.5)	8.93	0.002*
Education < high school	64 (29.8)	87 (60.0)	7.65	0.004*
Living alone	42 (19.5)	57 (39.3)	9.41	0.002*

The role of medication regimen complexity was further analyzed to determine whether it was associated with polypharmacy (≥ 5 medications) or specific drug regimens. Among non-adherent patients citing regimen complexity as a barrier, 67% (12/18) were on polypharmacy, compared to 32% (41/127) of other non-adherent patients, demonstrating a significant association (χ² = 6.88, p = 0.009). This suggests that patients taking multiple medications were more likely to experience difficulties in managing their treatment schedules. Additionally, beta-blockers and diuretics were disproportionately reported in patients citing regimen complexity compared to those on other antihypertensive drug classes (χ² = 5.71, p = 0.017). These findings highlight the need for medication reconciliation programs, simplified dosing regimens, and patient education to improve adherence. These findings are summarized in Table 5.

**Table 5:** Medication regimen complexity and non-adherence Statistical test conducted: Chi-square test. *A p-value < 0.05 is considered statistically significant.

Factor	Non-adherent patients citing regimen complexity, n (%)	Other non-adherent patients, n (%)	Chi-square (χ²)	p-value
Polypharmacy (≥5 medications)	12 (67.0)	41 (32.0)	6.88	0.009*
Beta-blockers	9 (50.0)	28 (22.0)	5.71	0.017*
Diuretics	8 (44.4)	24 (18.9)	5.71	0.017*

Cultural and religious beliefs also played a role in non-adherence, with 14 patients (9.7%) citing them as a barrier. A deeper analysis revealed that fasting during Ramadan was a primary concern, with nine patients (64.3%) reporting that they skipped or adjusted medication doses during fasting periods. Additionally, five patients (35.7%) expressed a preference for traditional medicine, believing herbal or alternative treatments were safer than prescribed medications. These insights suggest that targeted counseling during religious periods and educational interventions on the importance of continuous medication adherence could be beneficial in addressing these concerns.

Adherence rates varied significantly across the three hospitals included in the study, suggesting that institutional, demographic, and socioeconomic factors may influence patient adherence to antihypertensive treatment. Punjab Rangers Teaching Hospital had the highest adherence rate, with 119 out of 180 patients (66.1%) adhering to their prescribed regimen, while Sharif Medical and Dental College had an adherence rate of 58 out of 99 patients (58.7%), and Lady Reading Hospital had the lowest adherence rate: only 38 out of 81 patients (52.3%). The overall differences in adherence rates across hospitals were statistically significant (p < 0.05). A detailed breakdown of adherence rates by hospital is presented in Table 6.

**Table 6:** Hospital-wise adherence rates Statistical test conducted: Chi-square test. *A p-value < 0.05 is considered statistically significant.

Hospital	Adherent, n (%)	Non-adherent, n (%)	Total, n (%)	p-value
Punjab Rangers Teaching Hospital	119 (66.1)	61 (33.9)	180 (50.0)	0.021*
Sharif Medical and Dental College	58 (58.7)	41 (41.3)	99 (27.5)	0.035*
Lady Reading Hospital	38 (52.3)	43 (47.7)	81 (22.5)	0.044*
Total	215 (59.7)	145 (40.3)	360 (100)	-

Several factors could explain the significant variation in adherence rates across hospitals, including differences in patient demographics, socioeconomic conditions, and healthcare accessibility. Punjab Rangers Teaching Hospital had a greater proportion of urban residents, who typically have better access to healthcare resources, compared to Lady Reading Hospital, which had more patients from semi-urban or rural areas. Socioeconomic disparities also played a role, as patients at Lady Reading Hospital had lower average incomes and educational levels, both of which have been associated with poorer adherence. Financial constraints were a frequently cited reason for non-adherence at this hospital, with nearly half of non-adherent patients reporting difficulty affording medications. In contrast, Punjab Rangers Teaching Hospital had a higher proportion of insured patients and access to subsidized medications, which may have contributed to higher adherence rates.

Healthcare accessibility and hospital policies also influenced adherence. Punjab Rangers Teaching Hospital had structured patient counseling programs, better medication availability, and a digital follow-up system, all of which supported medication adherence. In contrast, Sharif Medical and Dental College and Lady Reading Hospital had less frequent adherence reinforcement programs, and medication stockouts were more commonly reported at Lady Reading Hospital. Physician-patient interaction and the frequency of follow-up visits may have also played a role, as Punjab Rangers Teaching Hospital emphasized routine follow-ups to monitor adherence.

To adjust for potential confounders, a multivariate logistic regression was performed, controlling for age, comorbidities, monthly income, education level, and urban vs. semi-urban residence. Even after adjustment, Punjab Rangers Teaching Hospital remained significantly associated with higher adherence (Adjusted OR = 1.84, p = 0.003). Lower education levels and low income independently predicted non-adherence, suggesting that socioeconomic barriers persist across all hospitals. Medication regimen complexity, particularly polypharmacy, was also a significant predictor of non-adherence, indicating that patients on multiple medications faced additional challenges in maintaining adherence.

These findings suggest that adherence rates are influenced by a combination of institutional policies, socioeconomic factors, and healthcare accessibility. While patient-related factors such as income and education play a role, hospital-specific interventions, such as structured adherence counseling, better medication availability, and improved follow-up systems, are crucial in improving adherence. Standardizing these interventions across hospitals could help reduce disparities in hypertension management and improve patient outcomes.

Psychosocial factors, including perceived stress, social support, and health literacy, had a significant impact on medication adherence. Non-adherent patients demonstrated higher levels of perceived stress, lower social support, and poorer health literacy compared to adherent individuals. These trends are detailed in Table 7.

**Table 7:** Psychosocial determinants of non-adherence Statistical test conducted: Chi-square test. *A p-value < 0.05 is considered statistically significant. DASS-21: Depression, Anxiety, and Stress Scale-21, MSPSS: Multidimensional Scale of Perceived Social Support, LMQ: Living with Medicines Questionnaire

Psychosocial factor	Adherent, n (%)	Non-adherent, n (%)	p-value
Perceived stress (DASS-21)			
Normal (0-13)	115 (53.5)	15 (10.3)	<0.001*
Mild (14-20)	60 (27.9)	23 (15.9)	
Moderate (21-27)	34 (15.8)	70 (48.3)	
Severe (28-33)	6 (2.8)	51 (35.9)	
Extremely severe (34+)	0 (0.0)	6 (4.1)	
Social support (MSPSS)			
Low (12-24)	64 (29.8)	62 (42.8)	<0.001*
Moderate (25-48)	104 (48.4)	54 (37.2)	
High (49-72)	47 (21.8)	29 (20.0)	
Health literacy (LMQ)			
Low	88 (40.9)	79 (54.5)	<0.001*
Moderate	97 (45.0)	47 (32.4)	
High	30 (14.1)	19 (13.1)	

To examine whether there was a linear association between increasing stress levels and non-adherence, a Spearman correlation test was conducted. The results confirmed a significant positive correlation between higher perceived stress scores and non-adherence (Spearman’s rho = 0.46, p < 0.001), indicating that as stress levels increased, adherence declined. Additionally, a Cochran-Armitage test for trend demonstrated a statistically significant increasing trend in non-adherence across worsening stress categories (p < 0.001). These findings suggest that stress may not only be a contributing factor but also a progressive determinant of non-adherence, warranting further exploration of stress management interventions in hypertensive patients.

Regarding social support, the moderate support category had a relatively high proportion of both adherent (48.4%) and non-adherent (37.2%) patients. A post-hoc pairwise comparison was conducted using Bonferroni-adjusted z-tests to assess differences between high vs. moderate and moderate vs. low social support levels. The results indicated that low social support was significantly associated with non-adherence when compared to moderate (p = 0.004) and high social support levels (p < 0.001). However, moderate vs. high social support did not significantly differ in their impact on adherence (p = 0.271). This suggests that while low social support is a strong predictor of non-adherence, patients with moderate and high levels of support may not exhibit substantially different adherence behaviors. These findings highlight the need for targeted interventions focused on individuals with low social support, as improving their network of assistance may yield the most significant improvements in adherence.

To assess whether the observed association between health literacy and adherence was confounded by educational attainment, a multivariate logistic regression analysis was performed. The adjusted model accounted for education level, age, income, and comorbidities. The results showed that even after adjusting for education, low health literacy remained an independent predictor of non-adherence (adjusted OR = 1.74, 95% CI: 1.15-2.65, p = 0.009). Education level was also independently associated with non-adherence (adjusted OR = 0.68, 95% CI: 0.46-0.98, p = 0.037), suggesting that both health literacy and formal education contribute to medication adherence, albeit through possibly distinct mechanisms.

## Discussion

The present study highlights the psychosocial determinants influencing non-adherence to antihypertensive therapy among patients in Pakistan. These findings indicate that low social support, higher perceived stress, and limited health literacy significantly contribute to poor adherence. In line with previous research, this study underscores the importance of non-biomedical factors in medication adherence, further advocating patient-centered strategies to enhance treatment compliance [19,20].

A significant association was observed between lower educational attainment and non-adherence, with patients with less than a high school education showing a markedly higher likelihood of poor adherence. Similar findings have been reported in studies from LMICs where limited health literacy reduces medication comprehension and adherence [21]. A systematic review highlighted that individuals with low health literacy are more likely to misunderstand prescription labels and dosing instructions, leading to decreased adherence [22]. Patients with lower educational levels may also have inadequate knowledge about hypertension complications, leading to a diminished perception of medication necessity [23]. These findings emphasize the need for tailored educational interventions to improve adherence, particularly in populations with restricted access to formal education.

The role of social support in medication adherence is another key finding of this study. Patients reporting lower scores on the MSPSS were significantly more likely to exhibit non-adherence. These results align with research demonstrating that family encouragement, peer involvement, and community support improve adherence rates [24,25]. The absence of a strong support system may contribute to forgetfulness, lack of motivation, or emotional distress, further reinforcing non-adherence behaviors. Implementing structured social support programs, counseling services, and patient engagement strategies could be beneficial for addressing these gaps.

Perceived stress was also found to be a strong determinant of non-adherence, as indicated by high scores on the DASS-21 [26]. Psychological stress has been linked to cognitive burden and emotional exhaustion, which can negatively impact self-management behaviors [27,28]. Previous studies have shown that stress-reducing interventions such as mindfulness training and behavioral therapy enhance medication adherence in chronic disease populations [29,30]. Given the psychological burden of chronic illnesses, addressing mental health in patients with hypertension may lead to improved adherence and overall disease management.

Financial constraints were another significant factor influencing non-adherence, with patients from lower-income backgrounds demonstrating a higher likelihood of skipping doses because of cost-related concerns. This finding is consistent with international studies that emphasize the financial barriers to medication adherence in LMICs [31]. High out-of-pocket expenditures for antihypertensive therapy exacerbate this problem, particularly in settings with limited health care subsidies. Studies indicate that financial burden is a critical determinant of adherence, as patients often prioritize basic living expenses over medication costs. Policymakers should consider subsidized medication programs, insurance coverage expansion, and cost-sharing initiatives to alleviate these financial burdens.

A comparison of adherence rates across different hospital sites revealed disparities, with patients from the Lady Reading Hospital demonstrating the lowest adherence levels. This variability may be attributed to differences in patient counseling, healthcare infrastructure, and resource availability, as previously noted in healthcare disparity research [32]. Institutional-level strategies, including enhanced pharmacist-led interventions and adherence-monitoring systems, may be instrumental in bridging these gaps. Reports on national healthcare disparities suggest that interventions tailored to specific institutional challenges, such as improved communication strategies and patient-centered care, can significantly improve adherence rates. Addressing these disparities through systematic reforms in hospital management and policy frameworks is essential to ensuring equitable access to healthcare resources.

Hypertension has emerged as a critical underlying factor contributing to catastrophic outcomes, including aortic dissection with rupture [33] and spontaneous intracerebral hemorrhage, both of which can lead to sudden death [34]. This study underscores the crucial role of psychosocial determinants in antihypertensive medication adherence, emphasizing that factors such as stress, socioeconomic conditions, and mental health significantly influence treatment compliance. Addressing these determinants is essential for improving medication adherence and, ultimately, reducing the risk of fatal cardiovascular and cerebrovascular events. These findings add a strong call to action for prevention strategies and public health interventions aimed at mitigating the devastating impact of hypertension-related complications.

Limitations

Although this study provides crucial insights into the psychosocial barriers affecting medication adherence, it is essential to consider its limitations. First, its cross-sectional design precludes causal inference, limiting the ability to establish temporal relationships between psychosocial determinants and adherence. Although we observed strong associations, these factors may act as mediators influenced by broader socioeconomic conditions. Future studies should consider longitudinal designs to establish causality better. Second, adherence was assessed using the self-reported MAQ, which, while validated, may introduce recall and social desirability bias. Future studies should consider objective adherence measures such as pharmacy refill records or electronic monitoring systems. Lastly, the study was conducted in tertiary care hospitals, potentially limiting its generalizability to primary healthcare settings and rural populations. Future research should explore adherence patterns in diverse healthcare settings to better inform intervention strategies.

## Conclusions

This study highlights the critical role of psychosocial determinants in adherence to antihypertensive medications. Low education level, inadequate social support, high perceived stress, and financial constraints were identified as key contributors to non-adherence. These findings underscore the need for targeted interventions, including patient education, psychosocial support mechanisms, and financial assistance programs, to enhance adherence rates.

From a clinical perspective, incorporating mental health screening and social support assessments into routine hypertension management may significantly improve patient outcomes. Future research should focus on longitudinal studies to assess the long-term impact of these interventions and explore innovative behavioral and digital health strategies to improve medication adherence in diverse patient populations. Additionally, pharmacist-led interventions such as structured medication counseling, short message service (SMS)-based reminder systems, and prescription cost assistance programs should be integrated into healthcare policies to enhance adherence in resource-limited settings.
